# BK002 Induces miR-192-5p-Mediated Apoptosis in Castration-Resistant Prostate Cancer Cells *via* Modulation of PI3K/CHOP

**DOI:** 10.3389/fonc.2022.791365

**Published:** 2022-03-07

**Authors:** Moon Nyeo Park, Hyunmin Park, Md. Ataur Rahman, Jeong Woo Kim, Se Sun Park, Yongmin Cho, Jinwon Choi, So-Ri Son, Dae Sik Jang, Bum-Sang Shim, Sung-Hoon Kim, Seong-Gyu Ko, Chunhoo Cheon, Bonglee Kim

**Affiliations:** ^1^ Department of Pathology, College of Korean Medicine, Kyung Hee University, Seoul, Republic of Korea; ^2^ Korean Medicine-Based Drug Repositioning Cancer Research Center, College of Korean Medicine, Kyung Hee University, Seoul, Republic of Korea; ^3^ Collage of Science in Pharmacy, Kyung Hee University, Seoul, Republic of Korea

**Keywords:** BK002, *Achyranthes japonica* Nakai, *Melandryum firmum* Rohrbach, castration-resistant prostate cancer, miR-192-5p

## Abstract

BK002 consists of *Achyranthes japonica* Nakai (AJN) and *Melandrium firmum* Rohrbach (MFR) that have been used as herbal medicines in China and Korea. AJN and MFR have been reported to have anti-inflammatory, anti-oxidative, and anti-cancer activities, although the synergistic targeting multiple anti-cancer mechanism in castration-resistant prostate cancer (CRPC) has not been well reported. However, the drug resistance and transition to the androgen-independent state of prostate cancer contributing to CRPC is not well studied. Here, we reported that BK002 exerted cytotoxicity and apoptosis in CRPC PC3 cell lines and prostate cancer DU145 cell lines examined by cytotoxicity, western blot, a LIVE/DEAD cell imaging assay, reactive oxygen species (ROS) detection, quantitative real-time polymerase chain reaction (RT-PCR), and transfection assays. The results from our investigation found that BK002 showed more cellular cytotoxicity than AJN and MFR alone, suggesting that BK002 exhibited potential cytotoxic properties. Consistently, BK002 increased DNA damage, and activated p-γH2A.X and depletion of survivin-activated ubiquitination of pro-PARP, caspase9, and caspase3. Notably, live cell imaging using confocal microscopy found that BK002 effectively increased DNA-binding red fluorescent intensity in PC3 and DU145 cells. Also, BK002 increased the anti-proliferative effect with activation of the C/EBP homologous protein (CHOP) and significantly attenuated PI3K/AKT expression. Notably, BK002-treated cells increased ROS generation and co-treatment of N-Acetyl-L-cysteine (NAC), an ROS inhibitor, significantly preventing ROS production and cellular cytotoxicity, suggesting that ROS production is essential for initiating apoptosis in PC3 and DU145 cells. In addition, we found that BK002 significantly enhanced miR-192-5p expression, and co-treatment with BK002 and miR-192-5p inhibitor significantly reduced miR-192-5p expression and cellular viability in PC3 and DU145 cells, indicating modulation of miR-192-5p mediated apoptosis. Finally, we found that BK002-mediated CHOP upregulation and PI3K downregulation were significantly reduced and restrained by miR-192-5p inhibitor respectively, suggesting that the anti-cancer effect of BK002 is associated with the miR-192-5p/PI3K/CHOP pathway. Therefore, our study reveals that a combination of AJN and MFR might be more effective than single treatment against apoptotic activities of both CRPC cells and prostate cancer cells.

## Introduction

Prostate cancer (PC) is a malignant cancer that represents the second highest death rate of male cancer worldwide, with 1.3 million new cases and 359,000 mortalities in 2018 (3.8% of cancer deaths) (1, 2). Prostate cancer can become castration-resistant prostate cancer (CRPC) after recurrence due to hormone deprivation therapy (3). CRPC is classified by intracrine/paracrine androgen secretion due to resistance acquired after testosterone deprivation therapy (4). The incidence of CRPC was estimated at 42,970 in 2020 and with annual progression in the US (5). However, the mortality rate rapidly progressed to a 50% rise in CRPC (6). After being diagnosed with CRPC, the survival rate is 9 to 13 months (7). Despite novel drugs, the mortality rate of CRPC is still high (8). The androgen receptor (AR) is stimulated by androgen binding including testosterone and dihydro testosterone which are responsible for development or reproductive function. However, 90% of the early stage of prostate cancer are AR-dependent. Many researchers had conducted studies for novel therapies for CRPC by 2020 (5). To date, abiraterone acetate (AA, Zytiga) and enzalutamide (Xtandi) are hormone inhibitors that have been approved by the Food and Drug Administration (FDA) for treatment of CRPC. Additionally, AA is an inhibitor that plays a role in inhibition of cytochrome P450 enzymatic activation associated with testosterone synthesis. Consistently, enzalutamide is responsible for the agonistic effect related to inhibiting by interfering with the translocation to the nucleus by competitively binding to AR. That is why the necessity of discovering a new biomarker has increased for CRPC (6).

Among the most three most well-known genes of prostate cancer, PI3K, RB, and RAS/RAF, here we investigated PI3K which is known to induce PTEN alteration and exert malignant progression in prostate cancer (9). Notably, PI3K is a major mediator of resistance to therapy in a wide range of alterations such as aggressive oncogene amplification as well as tumor suppressor deletion which lead to CRPC (10–13). Additionally, the endoplasmic reticulum (ER) plays a major role in protein synthesis and maturation which is known to be associated with disease and cancer due to its unfolded protein response (UPR) (14–17). Therefore, ER stress induced by cancer obviously promotes resistance to chemotherapy (18), and ER stress can reduce the apoptotic pathway by elevating the level of proliferation signaling activator PI3K (19, 20). Thus, in our study, we analyzed whether the BK002 contains ecdysterone related to drug resistance in cancer via modulation of PI3K and ER stress-induced ROS generation.

Currently, herbal medicine is known to cure the imbalance of the human body which causes diverse diseases including diabetes, neurodegenerative disease, and cancer (21, 22). To emphasize these, Korean traditional medicine are used as a complementary and alternative medicine (CAM) to modulate cancer (23, 24). Advantages of herbal medicine include less cytotoxicity, a reduction of side effects, and an increase in the effect of chemotherapy (24). Recently, ecdysteroids derived from plants have been reported to inhibit drug resistance in multidrug resistance (MDR) cancer cells (25–29). Recently, numerous researchers have investigated whether the anti-cancer mechanisms of traditional herbal medicines are related to the regulation between miRNA and cancer (30). In malignant hematological cancer, Spatholobus suberectus Dunn, Salvia miltiorrhiza, and Cnidium officinale Makino showed an anti-cancer effect via regulation of miR-657/ATF-2, miR-216b/c-Jun, and miR-211/CHOP, respectively (16a) (31, 32). MiR-192-5p has been found to have a potential anti-cancerous effect in lung cancer cells (33). *Achyranthes japonica* Nakai (AJN) was used for urinary problems including dysuria. *Melandrium firmum* Rohrbach (MFR) was also used as a traditional medicine for urinary problems, and tumor and blood stasis. This combination of AJN and MFR (known as BK002) is designed to increase the effect and reduce the side effects by using the two drugs at a low dose. Thus, in our study, the anti-cancer mechanism of BK002 treatment is investigated in androgen-independent prostate cancer cells through enhancing pro-apoptotic protein CHOP *via* downregulation of PI3K, AKT, and PARP. Additionally, we investigate whether BK002 anti-cancer and apoptosis effects are related to the miR-192-5p-mediated pathway in PC3 and DU145 prostate cancer cells.

## Materials and Methods

### Materials

MFR and AJN (200 g) each were harvested in Hongchungun, Gangwondo, Korea. We prepared the extracts as previously defined (34, 35). In brief, MFR, and AJN were filtered and extracted twice in 99% ethanol for 3 days each. The solution was extracted by an evaporator (EYELA, Yamato, Tokyo, Japan) and dried under a vacuum in freezing conditions (EYELA, Yamato, Tokyo, Japan). After extraction, the powder was dissolved in DMSO.

### High-Performance Liquid Chromatography Analysis for β-Ecdysterone

The ethanol extracts of MFR (50 mg) and AJN (20 mg) were dissolved in 1.0 and 2.0 ml of methanol, respectively, and sonicated for 1 h at room temperature. The standard solution of β-ecdysterone (SigmaAldrich, St. Louis, MO, USA) was prepared in methanol (0.5 mg/ml). To prepare the calibration standards, the standard solution was serially diluted and finally adjusted to 15.125, 31.25, 62.5, 125, and 250 μg/ml. Prior to HPLC analysis, samples and standard solutions were filtered with 0.2 μm PTFE filter (Whatman Inc., Maidstone, UK). The analysis was performed by Waters HPLC systems (Waters, Milford, MA, USA) equipped with the W1525 binary pump, W717 plus auto-sampler, and W996 PDA detector. The column was a Gemini NX C-18 110A column (5 μm, 250 x 4.6 mm I.D., Phenomenex International, USA). The flow rate was 0.7 ml/min with the mobile phase for aqueous 0.1% (v/v) trifluoroacetic acid (solvent A) and acetonitrile (solvent B). The linear gradient elution was as follows: 0–2 min, 10% B; 2–10 min 15% B; 10–40 min 25% B; 40–48 min 100% B; 48–49 min 5% B, and then 6 min to stabilize in the initial condition. The injection volume was 10.0 μL and the detection was conducted at 260 nm. All analysis was repeated three times to check its reproducibility.

### Cell Culture

PC3 (castration-resistant prostate cancer cell line) and DU145 (castration-resistant prostate cancer cell line) were purchased from ATCC. MDBK (normal kidney cell line) was obtained from Korean Cell Line Bank (KCLB, Seoul, Republic of Korea). The DU145 or PC3 cells, and MDBK cells were cultured with RPMI 1640 medium containing 10% fetal bovine serum (FBS), 2 μM of L-glutamine, and 10,000 U/ml of penicillin/streptomycin (Gibco, Grand Island, NY, USA). The medium was changed every 2-3 days.

### Cytotoxicity Assay

PC3, DU145, and MDBK cells were subjected to a cytotoxicity assay using an EZ-Cytox cell viability assay kit (Daeil Lab Service, Seoul, Republic of Korea) according to the manufacturer’s protocol. Cells were seeded in a 96-well plate in which various concentrations (12.5, 25, 50, 100, 200 μg/ml) of AJN and MFR were added for 24 h. The combination concentrations were determined as AJN (100 μg/ml) and MFR (50 μg/ml) in PC3 cells or AJN (50 μg/ml) and MFR (25 μg/ml) in DU145 cells for 24 h. The highest concentrations (0.035, 0.07, 0.15, 0.3, 0.6 μg/ml) of β-ecdysterone were determined in 200 μg/ml of AJN compared to MFR in PC3, DU145, and MDBK cells for 24 h. The absorbance values of cell viability were measured at 450 nm using a micro plate reader (Bio-Rad, Hercules, CA, USA).

### Western Blot Analysis

The protein was isolated from cells with lysis buffer (pH=7.4, 1% NP-40, 1 mM Na_3_VO_4_, 1 M EDTA, 1 mM NaF, 50 mM Tris-Hcl, 0.25% sodium deoxycholic acid, 150 mM NaCl) containing protease inhibitor cocktail (Amresco, Scolon, OH, USA). Protein quantification was normalized with β-actin using a Bio-Rad DC protein assay kit II (Bio-Rad, Hercules, CA, USA). The differences of protein expression were determined by Western blotting using SDS-PAGE 8% and 10% gel by electrophoresis. After blocking in 3% skim milk, the membrane with protein was probed with various primary antibodies for p-AKT, pro-PARP, CHOP (Cell signaling, Beverly, MA, USA), PI3K, and β-actin (Santa Cruz Biotechnologies, Santa Cruz, CA, USA) for 24 h followed by exposure to horseradish peroxidase (HRP)-conjugated secondary anti-mouse or rabbit antibodies for 1 h. Protein expression levels were identified by the chemiluminescence (ECL) system (Amersham Pharmacia, Piscataway, NJ, USA).

### Live and Dead Cell Imaging Assay

PC3 and DU145 (2×10^5^ a 6-well plate at 1 ml/well. At 24 h after seeding, the culture medium was treated with BK002 for 24 h. Cells were washed with DPBS and then loaded for 30 min with Calcein-AM green (LIVE/DEAD^®^ Viability/Cytotoxicity kit, Thermo Fisherscientific, Waltham, MA, USA) or ethidium homodimer-1 (LIVE/DEAD^®^ Cell imaging kit, Thermo Fisherscientific, USA) and added to each slide according to the manufacturer’s protocol. The images were obtained by confocal microscopy using FV10i (OLYMPUS Fluoview USA. Green: live cell; Red: dead cells; 50×, scale bar; 100 μm).

### Measurement of ROS

The Reactive Oxygen Species Detection Assay (Abcam, Cambridge, United Kingdom) was used to detect hydroxyl, peroxyl, and to analyze other ROS of cellular cytosolic hydrogen peroxide (H_2_O_2_). PC3 cells and DU145 cells were seeded into a 96-well plate and pretreated with N-Acetyl-L-cysteine (NAC, Sigma Aldrich Co., St. Louis, MO, USA) for 30 min; untreated NAC cells were also added. After being stained with 20 µM of DCFDA for 2 h in the dark at room temperature (RT), PC3 cells were treated with AJN (100 μg/ml) and MFR (50 μg/ml) and DU145 cells were treated with AJN (50 μg/ml) and MFR (25 μg/ml) for 2 h. Then the 96-well plate was measured using an ELISA reader (Bio-Rad, Hercules, CA, USA) (Ex/Em= 485/535 nm).

### Quantitative Real-Time PCR Analysis

RNA was isolated using the RNeasy mini kit (EZ™ Total RNA Mini prep Kit, Enzynomics, Korea). The total RNA was reversed transcribed using the HB miRNA Multi Assay kit ™ System І (HeimBiotek, Seoul, Republic of Korea) according to the manufacturer’s protocol. PCR started at 95°C for 15 min, followed by 40 cycles at 95°C for 10 s, 60°C for 40 s, and finished by 40 s at 60°C in the last cycle. The relative miRNA fold change was normalized using standard C_t_ values of RNU6B (U6) (HeimBiotek, Korea). The miR-specific primer Hsa-miR-192-5p was designed and synthesized by HeimBiotek Company. Three experiments were performed and analyzed by means of the 2^-ΔCT^ method. RT-PCR was performed using the Light Cyber™ instrument (Roche Applied Science, Indianapolis, IN, USA).

### Transfection Assay

PC3 cells and DU145 cells were transfected with the miR192-5p inhibitor using ViaFect™ Transfection Reagent according to the protocol. After transfection for 48 h, in DU145, AJN (50 μg/ml) and MFR (25 μg/ml) and in PC3, AJN (100 μg/ml) and MFR (50 μg/ml) were treated for 24 h. The inhibitor oligo base type with the following 2’ O-Methyl RNA base was applied by HeimBiotek (Seoul, Repulbic of Korea).

### Statistical Analysis

Data were presented as means ± standard deviation (SD). Statistically significant differences between the control and BK002-treated group were calculated by Student’s t-test using Sigma plot version 12 software (SysTest Software Inc., San Jose, CA, USA). All experiments were performed in triplicate. The value of p < 0.05 was considered to represent a statistically significant difference.

## Results

### Identification and Quantification of β-Ecdysterone in MFR and AJN by HPLC

**Figure 1:**
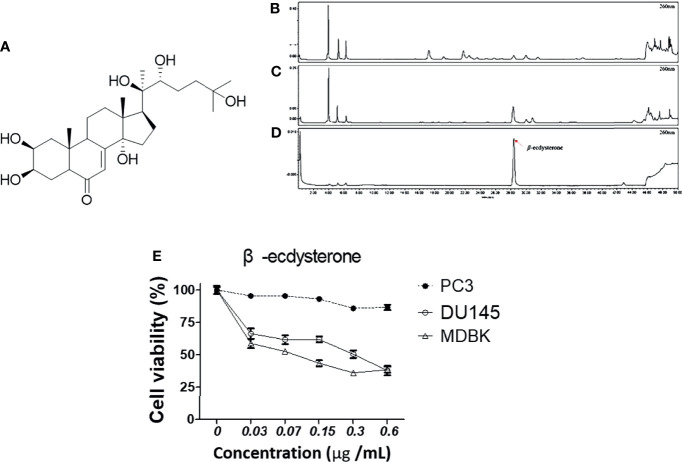
The HPLC chromatograms of **(A)** a diagram of the structure of β-ecdysterone **(B)** MFR, **(C)** AJN, and **(D)** β-ecdysterone detected at 260 nm. The presence of β-ecdysterone was confirmed according to the retention time and PDA spectrum. β-ecdysterone was observed at *R_t_* 28.36 min. **(E)** Cytotoxic effects of β-ecdysterone in PC3, DU145, and MDBK cells were determined in a concentration-dependent manner by an EZ-cytox cell viability assay. Data represent means ± SD; *p < 0.05, **p < 0.01, ***p < 0.001 compared to untreated control.

To check whether MFR and AJN contain β-ecdysterone, we measured β-ecdysterone level by HPLC methods. β-ecdysterone and phytoestrogen derived from the root of *A. bidentata* have been reported to be anti-oxidative in a concentration-dependent manner (36, 37). The HPLC chromatograms showed the existence of β-ecdysterone (*R_t_* = 28.36 min) in BK002 by comparison with retention time (*R_t_*) and photodiode array (PDA) spectrum of the standard solution (**Figure 1**). To quantify the content of β-ecdysterone, the calibration curve of the standard was obtained with serially diluted solutions (15–250 μg/ml). Here, we found that the MFR and AJN β-ecdysterone peak was very small (**Figures 1B–D**), additionally, β-ecdysterone exhibited more cellular cytotoxicity in normal MDBK cells than prostate cancer PC and DU145 cells (**Figure 1E**), suggesting that β-ecdysterone has no toxic effects on cancer cells. The regression equation was *y* = 16421*x* - 36509 (*r* ^2^ = 0.9993, n = 5). The contents of β-ecdysterone in MFR and AJN were determined to be 66.43 ± 2.82 and 307.59 ± 4.18 mg/100g, respectively (**Table 1**).

**Table 1:** Calibration curve of β-ecdysterone.

Standard solutions		Regression equations		R^2 8^	Stability (RSD%)			Inter-day		Intra-dav	
*β*-ecdysterone		y = 16421x + 36509		0.9993	0.155053			RT^3^ (mm)	RSD^4^	RT^3^ (mm)	RSD^4^
						*β*-ecdysterone		28.38083	0.155053	28.41133	0.389274
	Area.	ppm^6^	Sample conc.	*β* -ecdysterone						*β*-ecdysterone	
				Conc. (%)^7^	Conc.(mg/100g)^7^		Area.	ppm^6^	Sample conc.	Conc. (%)^7^	Conc.(mg/100g)
MFR^1^	584.765	33.213	50 mg ml	0.0664260	66.43	AJN^2^	1.301,194	76.898	25 mg/ml	0.3075933	307.59
SD^5^	23.098	1.408		0.003	2.817	SD^5^	17.122	1.044		0.004	4.178

All the results were shown as the mean n = 3.

^1^MFR, Melandrium firmum Rohrbach; ^2^AJN, Achyranthes japonica Nakai; ^3^RT, Retention time;^4^ RSD, relative standard deviations; ^5^ SD, standard deviation; ^6^ppm, parts per million; ^7^Con., concentration; ^8^R^2^, R-squared.

### AJN and MFR Exerts Cytotoxicity in PC3 and DU145 Prostate Cancer Cells

**Figure 2:**
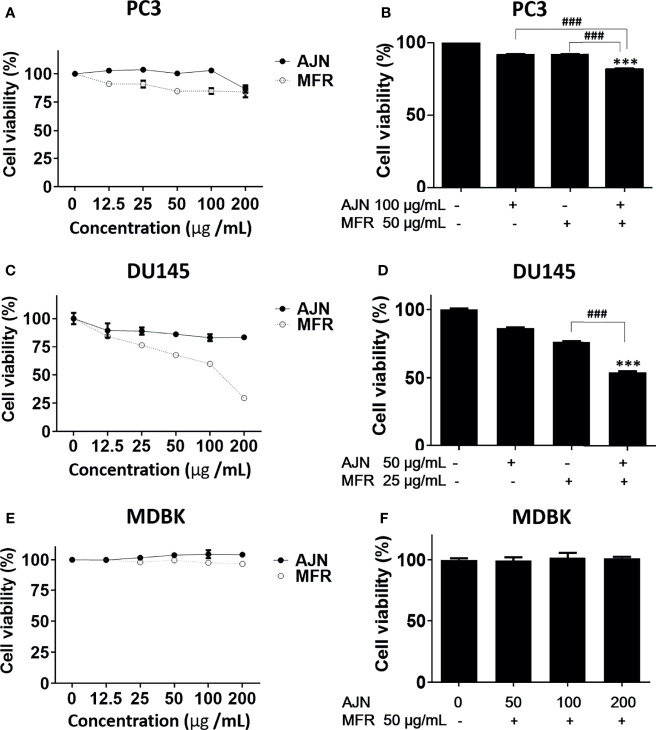
Cytotoxic effects of AJN and MFR in PC3 and DU145 cells. The indicated concentrations of AJN and MFR were added to **(A)** PC3, **(C)** DU145, and **(E)** MDBK for 24 h. **(B, D, F)** A cell viability assay was performed in AJN and MFR-treated cells using an EZ-cytox cell viability assay. The values above represent the means of three experiments. Means ± SD; ***p<0.001 compared to untreated control, ^###^ *p*<0.001 between two groups.

To investigate the potency of BK002 against prostate cancer cells, the different concentrations of AJN, MFR, and BK002 were exposed to PC3 and DU145 cells for 24 h, and the numbers of viable cells were determined by an EZ-Cytox cell viability assay. In the results from our study, we found that AJN and MFR concentration dependently reduced cellular viability in PC3 prostate cancer cells (**Figure 2A**). On the other hand, combination of AJN (100 µg/ml) and MFR (50 µg/ml), called BK002, significantly reduced more cellular viability than AJN and MFR single treatment in PC3 cells (**Figure 2B**). However, in DU145 cells, AJN and MFR concentration dependently reduced cell viability and combination of AJN (50 µg/ml) and MFR (25 µg/ml) significantly decreased more cellular viability than AJN and MFR single treatment (**Figures 2C, D**). On the other hand, AJN, MFR, and BK002 did not show any cytotoxic effect on normal MDBK cells (**Figures 2E, F**). Taken together, these results indicate that BK002 was more effective and cytotoxic in prostate cancer cells rather than normal cells which suggests the combined use of AJN and MFR for more cytotoxic activities than single AJN and MFR in our further investigations.

### AJN and MFR Exhibit Anti-Proliferative Effects in PC3 and DU145 Cells

**Figure 3:**
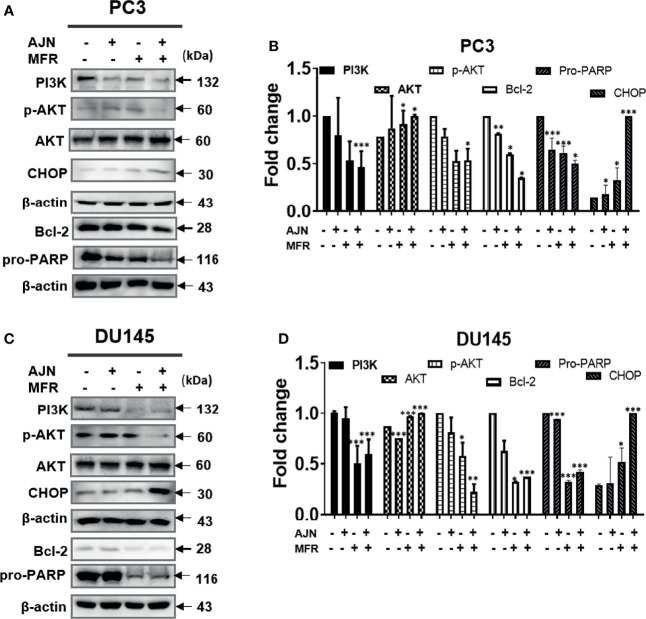
Treatment of BK002 induced apoptosis in PC3 and DU145 cells. Cells were treated with AJN (100 mg/ml) and MFR (50 mg/ml) in **(A)** PC3 cells or AJN (50 mg/ml) and MFR (25 mg/ml) in **(C)** DU145 cells for 24 h. The proteins isolated from both PC3 and DU145 cells were subjected to western blot for PI3K, p-AKT pro-PARP, and CHOP. β-actin was exploited as a loading control. **(B, D)** The graph shows the quantification of western blot replicates. Data represent means ± SD; *p<0.05, **p<0.01, ***p<0.001 compared to untreated control.

To further determine the cytotoxic effect of BK002 on PC3 and DU145 cells, we were interested to know whether these cytotoxic effects might be caused by induction of apoptotic mechanisms determined by western blot in addition to a live and dead cell assay. It has been found that dysregulation of anti-apoptotic proteins PI3K and AKT are known to be associated with CRPC progression as well as drug resistance (38, 39). Here, we found that single treatment of AJN and MFR exhibited lower PI3K and phosphor-AKT expression, on the other hand, combined AJN and MFR (BK002)-treated cells showed a significant reduction of PI3K and phosphor-AKT on PC3 and DU145 cells (**Figures 3A** **–D**). It has been implicated that pro-apoptotic protein CHOP, belonging to the family of CCAAT/enhancer binding proteins (C/EBPs), is involved in gene regulation of cellular proliferation, differentiation, and energy metabolism which plays a crucial role to induce apoptosis (40). Moreover, anti-apoptotic poly (ADP-ribosyl)ation of nuclear proteins (PARPs) has been shown to be required for apoptosis induction in various cell lines (41). In our investigation, we found that BK002-treated cells significantly attenuated the full length of pro-PARP and increased CHOP expression in DU145 cells better than in PC3 cells, indicating that cytotoxicity was enabled by an apoptosis-mediated pathway in both prostate cells (**Figures 3A** **–D**)

### BK002 Exhibits Apoptosis in PC3 and DU145 Cells

**Figure 4:**
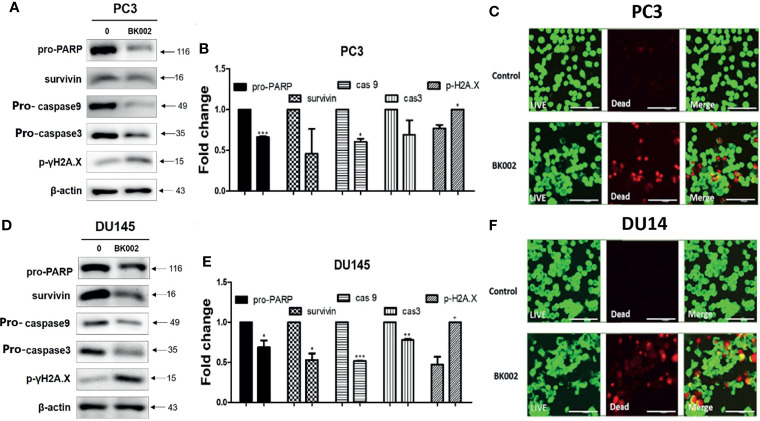
Treatment of BK002 induced apoptosis in PC3 and DU145 cells. Cells were treated with AJN (100 μg/ml) and MFR (50 μg/ml) in **(A–C)** PC3 cells or AJN (50 μg/ml) and MFR (25 μg/ml) in **(D–F)** DU145 cells for 24 h. **(A, D)** Effect of BK002 on pro-PARP, survivin, pro-caspase-9, pro-caspase-3, and p-γH2A.X in PC3 and DU145 cells. Both PC3 and DU145 cells were subjected to western blot analysis. **(B, E)** The bar graph represents the results from the western blot analysis. **(C, F)** Cells were stained with calcein AM and ethidium homodimer-1 for the live and dead assay. The green fluorescent indicates the live cells and red fluorescent indicates dead cells. Live and dead results were visualized with a fluorescent optical filter (485 ± 10 nm) and rhodamine optical filter (530 ± 12.5 nm). Magnification × 50. (Scale bar, 100 μm). The graph shows the quantification of western blot replicates. Data represent means ± SD; *p<0.05, **p<0.01, ***p<0.001 compared to untreated control.

To further investigate whether the anti-cancer effects of BK002 possibly lead to apoptosis, western blotting and a live and dead cell assay were performed in PC3 and DU145 cells. It is well known that drug resistance in association with the poor survival rate of the patient and apoptosis inhibition are modulated by survivin (42). To determine whether BK002 inhibited drug resistance and anti-apoptotic factors, western blotting was adopted in PC3 and DU145 cells. As shown in **Figures 4** **A, D**, the expression of survivin was suppressed compared to untreated groups. Consistently, activation of caspase was confirmed in BK002-treated PC3 and DU145 cells. Pro-PARP, pro-caspase 9, and pro-caspase 3 were depleted in PC3 and DU145 cells, compared to untreated groups (**Figures 4** **A, D**). In addition, BK002 increased DNA damage marker p- γH2A.X in PC3 and DU145 cells (**Figures 4** **A, D**). Similarly, it was confirmed that the red fluorescence probe was significantly increased due to dead cells in BK002-treated cells compared to the untreated group by confocal microscopy (**Figures 4C, F**).

### BK002 Promotes ROS Generation and ROS Scavenger Attenuates Cytotoxicity of BK002-Treated Prostate Cancer Cells

**Figure 5:**
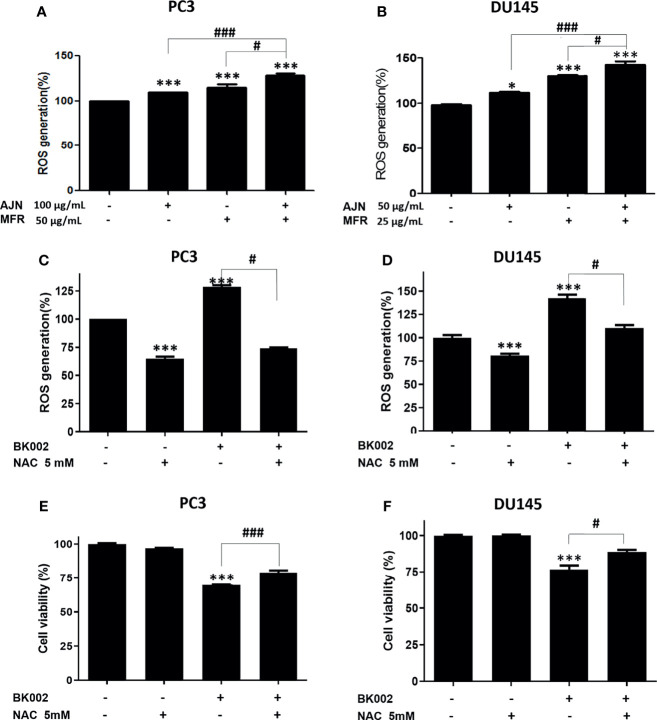
BK002 increased ROS generation and NAC pretreatment reduced the cytotoxic effect of BK002 in PC3 and DU145 cells. Cells were subjected to a permeable fluorescent-based and chemiluminescent probe with 20 μM of DCFDA for 45 min at 37 °C in the dark. Cells were treated with AJN (100 μg/ml) and MFR (50 μg/ml) in **(A)** PC3 cells or AJN (50 μg/ml) and MFR (25 μg/ml) in **(B)** DU145 cells for 24 h. ROS generation was measured by using a microplate reader. **(C, D)** NAC was pretreated, and the cytotoxic effect of BK002 was studied in PC and DU145 cells. **(E, F)** A cell viability assay was conducted using EZ-Cytox by an absorbance measurement *via* an optical spectrometer. (Ex/Em=485/535). The values above represent the means of three experiments. Means ± SD; *p<0.05, ***p<0.001 compared to untreated control, ^#^ *p*<0.05, ^###^ *p*<0.001 between two groups.

ROS has a critical role in cell death-related pathways due to severe ER stress (27). To evaluate the effect on ROS generation of BK002-mediated apoptosis, a ROS measurement was performed by a fluorescent-based 2’, 7’-dichlorofluorescein diacetate (DCFDA) assay. Here, we found that AJN and MFR treatment significantly increased ROS generation in both PC3 and DU145 cells (**Figures 5A, B**). In addition, combined treatment of AJN and MFR (BK002) significantly promoted more ROS generation compared to single AJN and MFR treatment in both PC3 and DU145 cells (**Figures 5A, B**). Thus, the results indicate that BK002-mediated apoptosis has an important role in generating ROS production in prostate cancer cells. To confirm the role of ROS production in BK002-induced apoptosis, we further used a ROS inhibitor, N-acetyl-L-cysteine (NAC) (43), and examined ROS generation by an ROS detection assay. Here, we found that BK002-treated cells significantly increased ROS generation, and co-treatment with NAC and BK002 significantly decreased ROS production in both PC3 and DU145 cells (**Figures 5C, D**). Concomitantly, the cytotoxic effect of BK002 was significantly restrained when pre-treated by NAC on both PC3 and DU145 cells (**Figures 5E, F**). Because of an error, there was a difference between the cytotoxicity value in **Figures 2D** and **Figure 5F**. Therefore, these observations suggest that BK002-mediated apoptosis might contribute to ROS generation in prostate cancer cells.

### BK002 Increases MicroRNA-192-5p Expression in Prostate Cancer Cells

**Figure 6:**
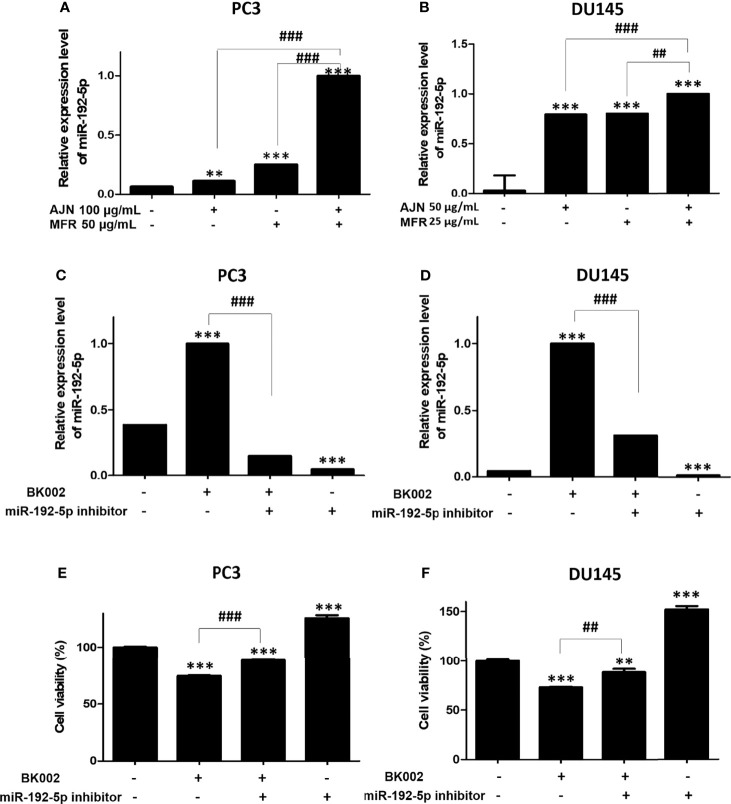
Treatment of BK002 significantly increased miR-192-5p expression in PC3 and DU145 cells. Cells were treated with AJN (100 μg/ml) and MFR (50 μg/ml) in **(A)** PC3 cells or AJN (50 μg/ml) and MFR (25 μg/ml) in **(B)** DU145 cells for 24 h and the expression of miR-192-5p was measured by qRT-PCR in PC3 and DU145 cells. **(C, D)** Cells were transfected with miR-192-5p inhibitor for 48 h using a transfection reagent and the expression of miR-192-5p was calculated. **(E, F)** The effect of BK002 on cell viability in miR-192-5p inhibitor-transfected PC3 and DU145 cells was determined by an EZ-CYTOX cell viability assay kit. The values above represent the means of three experiments. Means ± SD; **p<0.01, ***p<0.001 compared to untreated control, ^##^ *p*<0.01, ^###^ *p*<0.001 between two groups.

Recently, it has been found that miR-192-5p, a member of the miR-192 family, plays a crucial role in vital biological processes and regulates oxidative stress, proliferation, apoptosis, inflammatory responses, and various cancers such as lung, liver, and breast (44). To check the effect of BK002 on the expression of miR-192-5p, qRT-PCR was performed. We found that combined treatment of AJN and MFR significantly upregulated the expression of miR-192-5p compared to single AJN and MFR treatment in both PC3 and DU145 cells (**Figures 6A, B**). Additionally, co-treatment of BK002 and miR-192-5p inhibitor significantly decreased miR-192-5p expression in both PC3 and DU145 cells determined by a transfection assay (**Figures 6C, D**). However, cellular viability was significantly increased with co-treatment of BK002 and miR-192-5p inhibitor in both prostate cancer cells (**Figures 6E, F**). All together these investigations suggest that BK002-induced cytotoxicity is also dependent on a miR-192-5p-mediated pathway.

### BK002 Regulates Apoptosis-Related Protein *via* Modulation of miR-192-5p in Prostate Cells

**Figure 7:**
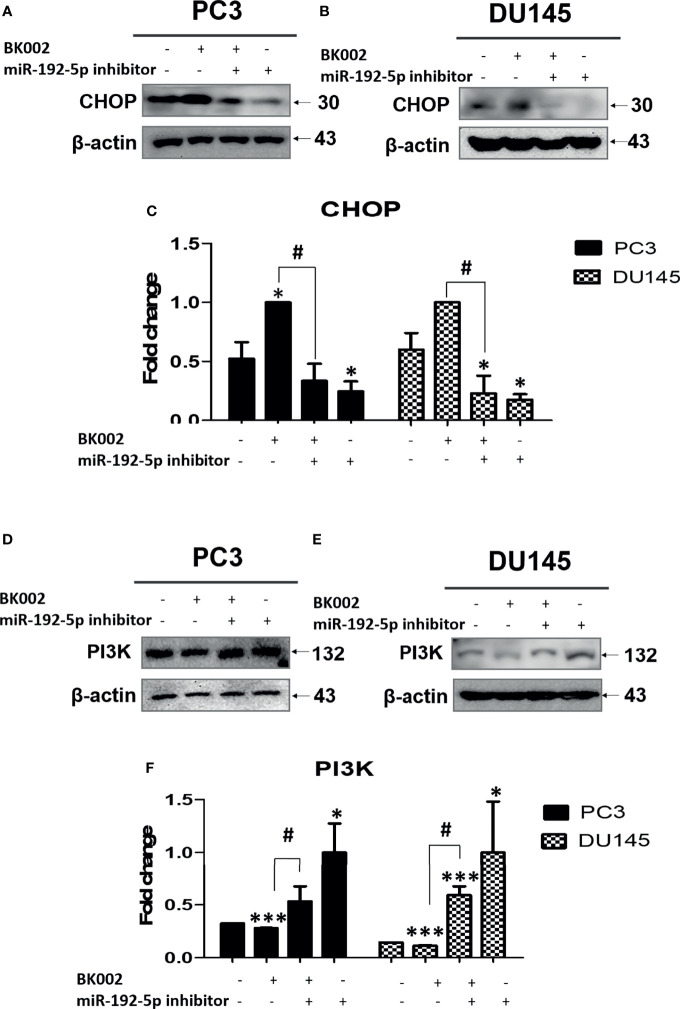
The anti-cancer effect of BK002 was inhibited by miR-192-5p inhibition. Cells were transfected with miR-192-5p inhibitor for 48 h using a transfection reagent, and the expression of miR-192-5p was calculated. Cells were treated with AJN (100 μg/ml) and MFR (50 μg/ml) in **(A, D)** PC3 cells or AJN (50 μg/ml) and MFR (25 μg/ml) in **(B, E)** DU145 cells for 24 h. Western blotting was conducted for CHOP and PI3K in PC3 and DU145 cells. **(C, F)** The bar graph represents the fold change of protein expression above. The values above represent the means of three experiments. Means ± SD; *p<0.05, ***p<0.001 compared to untreated control, ^#^p<0.05 between two groups.

We investigated whether the role of miR-192-5p in an apoptosis pathway and CHOP and PI3K expressions are closely related with cancer progression (45). Here, we found that co-treatment with BK002 and miR-192-5p inhibitor significantly reduced pro-apoptotic protein CHOP expression in both cells when miR-192-5p was suppressed by the transfection of miR-192-5p inhibitor (**Figures 7A–C**). Conversely, PI3K expression was significantly restrained with co-treatment of BK002 and miR-192-5p inhibitor in PC3 cells and DU145 cells examined by suppression of miR-192-5p *via* transfection of miR-192-5p inhibitor (**Figures 7D–F**). Taken together our results demonstrate that BK002-mediated apoptosis was regulated by miR-192-5p in prostate cancer cells.

## Discussion

Prostate cancer has the third highest mortality rate of middle and older aged men characterized by malignant progression due to frequently recurrence and resistance (46, 47). Approximately, 90% of cases have an increased survival rate with early treatment such as local radiotherapy, prostatectomy, and chemotherapy (47). Notably, over 30% of patients diagnosed with disease progression depend on androgen, at this state, androgen deprivation is a very effective treatment including single and combination administration of gonadotropin-releasing hormone (GnRH). Despite such a prognosis, most patients commonly experience recurrence within 3 years and the disease state progresses toward prostate cancer (48). In the present study, we investigated the anti-cancer effect of natural plant extracts on CRPC PC3 and DU145 prostate cancer cells and focused our mechanistic investigations on cancer treatment resistance as well as micro-RNA-192-5p modulation.

Prostate cancer is treated by androgen deprivation therapy (ADT), referred to as endocrine therapy, that leads to 80% symptomatic improvement, temporarily. Then, most patients experience a hormone-independent state. In such a condition, we should change the terminology from hormone-independent cancer to CRPC (49). CRPC was identified as the major cause of morbidity in prostate cancer (50). The treatment selection of the CRPC patient is restricted to docetaxel and prednisone (DP) which was approved by the United States’ FDA in 2004. The benefit of DP is not for improving the survival rate but consolidation of bone pain due to palliative quality of life, of which tolerability is still debatable due to the burden to older patients. During a 3-week trial, DP was replaced by mitoxantrone and prednisone (MP) as the standard guideline of care for CRPC (50). The median progression-free survival (PFS) of MP was about 6 months and overall survival (OS) was less than 2 years (50). Despite chemotherapy and consolidation treatment, patients had an unfavorable response due to acquired heterogenous mutations and different side effects (51). Recently, it has been found that natural products have been used as a complement to cancer chemotherapy *via* pharmacological modulation of the apoptosis pathway (52). Of note, natural products have been known to have antitumor, antioxidative, antibacterial, and anti-inflammatory capabilities as they contain bioactive components (53). Therefore, natural products might be required for further consideration of apoptosis induction which could lead to treatment for castration-resistant prostate cancer. Herein, a natural product is required for accurate analysis due to the diversity of bioactive components which lead to toxicity by accumulative doses containing components not yet identified (54). Recently, it was found that a combination of β-ecdysterone (250~750 μM) and doxorubicin (0.15 or 0.25 μM) enhanced the anticancer effect in drug-resistant breast cancer cells (55). Based on the above, to find the potential pharmacological effect of BK002 in CRPC, qualitative and quantitative analysis of bioactive compound β-ecdysterone in BK002 was investigated by HPLC (**Figure 1**). Thus, the underlying anticancer effect of BK002 was investigated in association with multiple targeting mechanisms for possible applications in CRPC.

Of note, we elucidated that combined treatment of *Achyranthes japonica* Nakai and *Melandrium firmum* Rohrbach (BK002) increased significant cytotoxicity in prostate cancer cells without killing normal cells, implying selective damage to cancer in association with a reduction of side effects (56, 57) (**Figure 2**). Additionally, for the possibility of a synergistic anti-cancer effect, single treatment compared to the combination of BK002 was investigated. Notably, the effect of BK002 was significant in PC3 and DU145 in prostate cancer cells without affecting MDBK normal cells. PC3 cells have a high aggressive metastatic potential and DU145 cells have a moderate aggressive potential that is consistent with the fact that DU145 cells are more susceptible to BK002 than PC3 cells. In addition, both PC3 and DU145 cells are similar to androgen hormone-independent cells.

Recently, analysis of accumulation of AS in CRPC has been reported, which is controlled by interconnection between the truncated isoform of AR activation and the PI3K pathway (58, 59). Accordingly, the PI3K pathway has been highlighted as a prognostic and clinical biomarker of CRPC (60, 61). Nevertheless, the underlying anticancer effect of BK002 has not been revealed in relation to regulation of PI3K in CRPC. To date, BK002 has been mostly used for anti-diabetic, anti-inflammatory, anti-microbial, anti-oxidative, and osteoprotective effect (62). In the present investigation, we found that BK002-induced apoptosis was associated with PI3K regulation in CRPC (**Figure 3**). Additionally, the ER implement retained the homeostasis of posttranslational modification for protein activity and structure (63). Whereas, the ER stress condition might be generated by a cancer-derived abnormal state including hypoxia and malnutrition, resulting in accumulations of unfolded proteins. Under severe ER stress, the ER-induced CHOP-mediated apoptosis pathway was associated with degradation of pro-PARP (50, 57). Besides, BK002 significantly reduced PI3K and phospho-AKT, a castration-resistant progression biomarker, and this apoptosis increased ER-related apoptotic proteins such as CHOP and pro-PARP (**Figure 3**). Therefore, in this study, the role of PI3K and CHOP has been elucidated in association with BK002-induced anti-cancer effect in CHOP-sensitive and hormone-independent PC3 cells and DU145 prostate cancer cells.

Moreover, DNA damage-induced apoptosis signaling is the critical target for cancer treatment (64). So far, survivin has been known as a poor prognostic factor in various malignant cancers due to chemoresistance and inhibition of caspase activation (42, 65). Herein, BK002-treated PC3 and DU145 cells significantly suppressed survivin leading to caspase activation. BK002 stimulated the inactive zymogenic form of caspase such as caspase 9 or caspase 3 or pro-PARP and was modified posttranslationally by ubiquitination (66) (**Figure 4**). It is well known that p-γH2A.X is a critical marker for double strand breaks (DSBs) due to ionizing radiation or chemotherapy (67). Herein, BK002 significantly induced the expression of p-γH2A.X, demonstrating potential pro-apoptotic properties in resistant prostate cancer cells and prostate cancer cells as well. Notably, this was confirmed by the LIVE/DEAD™ Cell imaging kit using dying cell DNA-binding dyes in BK002-treated PC3 cells or DU145 cells (**Figure 4**). Of note, fluorescein diacetate (FDA) is a very sensitive and selective probe that is associated with live cells, where green fluorescence is produced by cytoplasmic esterase, and in dying and dead cells, a bright red fluorescence is generated upon binding to DNA (68). Consistently, BK002 promoted effective apoptosis in DU145 cells as well as resistant PC cells, contributing to deepen the biological understanding under the live cell condition.

While studying whether the anticancer effect of BK002 is due to ER stress-related apoptosis, we found that BK002 significantly induced expression of ER-related apoptotic proteins such as CHOP and caspase activation (**Figures 3**, **4**).

Under normal conditions, ROS are responsible for stimulation of a second messenger in the Ca^2+^-mediated cascade due to mitochondrial oxidative respiration. Meanwhile, ER stress caused by the upsurge of ROS generation in cancer cells persists in ROS-mediated cancer cell death. Several natural products have been found to induce apoptosis-mediated cell death *via* modulation of ROS generation. For example, the antiproliferation effect of natural products from *Withania somnifera* encourages ROS generation in addition to mitochondria-induced apoptosis in HL-60 myeloid leukemia cells (69). BK002 triggered an upsurge of ROS generation compared to control and single AJN and MFR treatment. These results have been identified as similar in ROS mediated-apoptosis induced by natural compounds (70, 71). Furthermore, ROS generation is reduced by ROS inhibitor NAC, implying the anticancer effect in DU145 cells or PC3 cells in accordance with ROS-mediated apoptosis against CRPC (**Figure 5**). Therefore, the current study suggests that BK002-mediated apoptosis is required to generate ROS production.

It has been extensively investigated whether miRNAs function as oncogene silencers or tumor suppressor gene enhancers depending on the target mRNA in various cancers including colon, prostate, pancreatic, lung, breast, bladder, and kidney (72). Recently, herbal medicine has been identified as having a potential anti-cancer effect *via* regulation of the miRNA network (73). Several studies have revealed the anti-cancer effect of a Sophorae Flos and *Lonicerae japonicae* Flos-regulated miR-let-7/f-CCR7 network (74), SSD-regulated miR-657/ATF2 network (16), *SM-*regulated miR-216b/c-Jun network (32), COM extract-regulated miR-211/CHOP network (31), and *Panax ginseng* C.A. Meyer (Rg3)-regulated miR-21/PI3K/AKT network (75). Accumulated evidence has shown that the herbal medicine-derived component inhibited oncogenes or enhanced tumor suppressor genes (76). In the present investigation, we found that transfection of miR-192-5p inhibitor significantly repressed miR-192-5p and increased cell viability *via* co-treatment with BK002 (**Figure 6**). In addition, our investigation also demonstrated that the mature sequence containing hsa-miR-192-5p within miR-192 was a significantly repressed oncogene and induced CHOP-mediated ER stress-related apoptosis (**Figure 7**). MiR-192-5p has been identified as a poor prognostic factor in metastatic colon cancer (77, 78). Here, our results suggested that BK002 increased miR-192-5p which implied the potential regulation of apoptosis *via* the miR-192-5p/PI3K pathway.

## Conclusions

**Figure 8:**
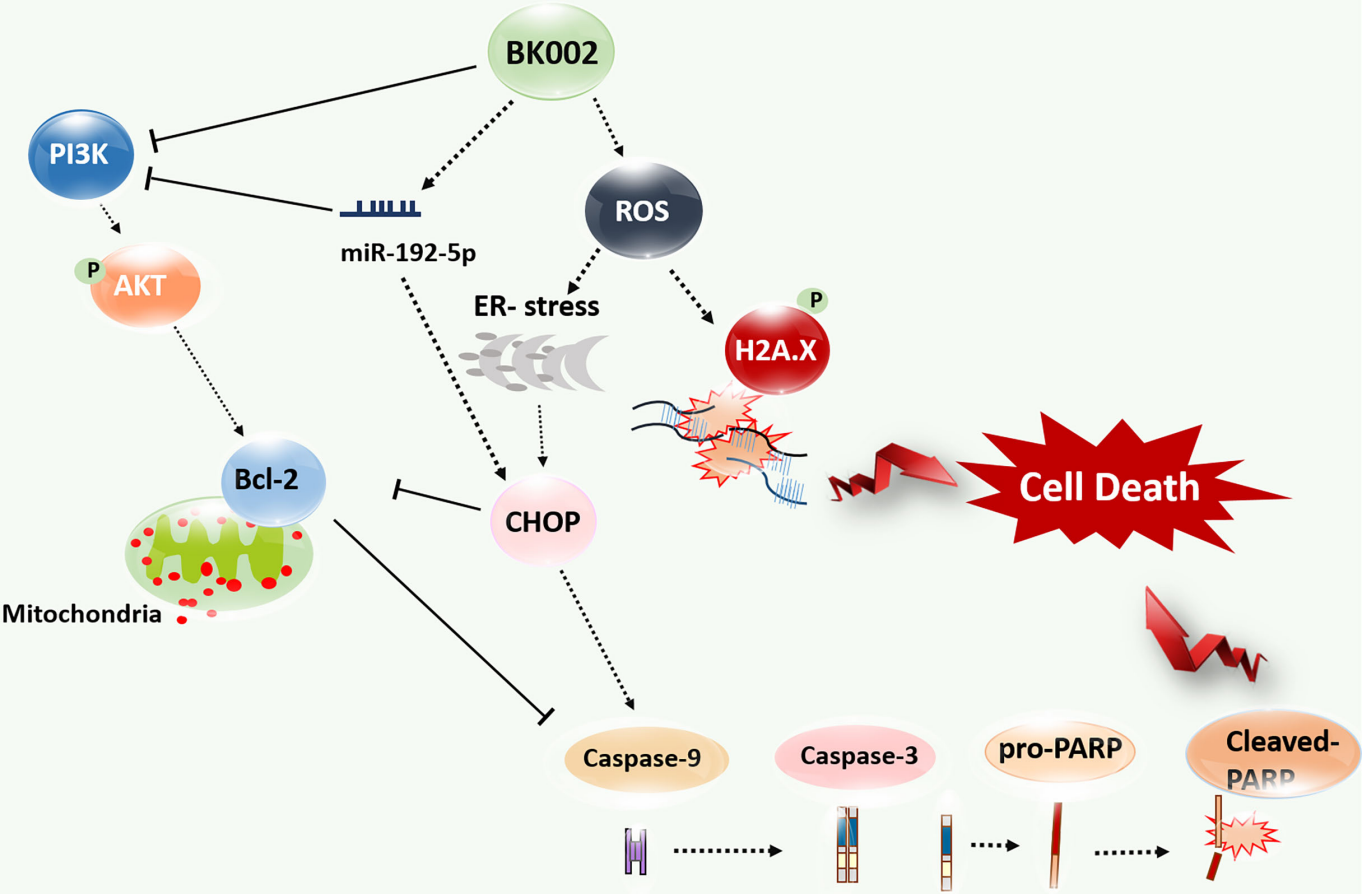
Model of BK002-mediated apoptosis induction in castration-resistant prostate cancer (CRPC) cells and prostate cancer cells. BK002 efficiently encouraged ROS generation which activated ER stress and CHOP in conjunction with apoptotic cascade caspase activation and activation of p-γH2A.X leading to apoptosis. PI3K, p-AKT, and survivin were subsequently downregulated by BK002 treatment in CRPC cells and prostate cancer cells to induce apoptosis. Moreover, BK002 increased miR-192-5p expression and miR 192-5p regulated apoptosis.

BK002 has been shown to have a significant effect on prostate cancer in PC3 cells and DU145 cells without affecting normal cells. Notably, BK002 treatment efficiently induced ROS-mediated endoplasmic reticulum-associated degradation (ERAD) in proteins such as CHOP along with caspase activation and attenuated survivin or PI3K/AKT, leading to activation of p-γH2A.X. Moreover, BK002 treatment upregulated miR-192-5p, and inhibition of miR-192-5p modulated apoptosis signaling through regulation of CHOP and PI3K. BK002-mediated apoptosis induction has been presented in our proposed model in **Figure 8**. Therefore, the present study suggests that BK002 synergistic treatment might be useful as a potential therapeutic approach in CRPC control compared to single treatment of *Achyranthes japonica* Nakai and *Melandrium firmum* Rohrbach.

## Data Availability Statement

The datasets presented in this study can be found in online repositories. The names of the repository/repositories and accession number(s) can be found in the article/supplementary material.

## Author Contributions

Conceptualization and writing—original draft preparation: MP, and HP. Formal analysis: CC, S-RS, and DJ. Data curation: JK, SP, YC, MR, and JC. Writing—review and editing: MP, HP, S-GK, B-SS, S-HK, MR, and BK. Visualization: MP and MR. Supervision: B-SS, S-HK, and BK. Project administration: BK. Funding acquisition: S-GK and BK. All authors have read and agreed to the published version of the manuscript.

## Funding

This research was supported by the Basic Science Research Program through the National Research Foundation of Korea (NRF) funded by the Ministry of Education (NRF-2020R1I1A2066868), the National Research Foundation of Korea (NRF) grant funded by the Korea government (MSIT) (No. 2020R1A5A2019413), a grant from the Korea Health Technology R&D Project through the Korea Health Industry Development Institute (KHIDI), funded by the Ministry of Health & Welfare, Republic of Korea (grant number: HF20C0116), and a grant from the Korea Health Technology R&D Project through the Korea Health Industry Development Institute (KHIDI), funded by the Ministry of Health & Welfare, Republic of Korea (grant number: HF20C0038).

## Conflict of Interest

The authors declare that the research was conducted in the absence of any commercial or financial relationships that could be construed as a potential conflict of interest.

## Publisher’s Note

All claims expressed in this article are solely those of the authors and do not necessarily represent those of their affiliated organizations, or those of the publisher, the editors and the reviewers. Any product that may be evaluated in this article, or claim that may be made by its manufacturer, is not guaranteed or endorsed by the publisher.
